# Dietary glycine supplementation enhances syntheses of creatine and glutathione by tissues of hybrid striped bass (*Morone saxatilis ♀* × *Morone chrysops ♂*) fed soybean meal-based diets

**DOI:** 10.1186/s40104-024-01024-5

**Published:** 2024-05-09

**Authors:** Wenliang He, Xinyu Li, Guoyao Wu

**Affiliations:** Department of Animal Science, Texas A&M University, College Station, TX 77843 USA

**Keywords:** Amino acids, Creatine, Fish, Glutathione, Glycine, Nutrition

## Abstract

**Background:**

We recently reported that supplementing glycine to soybean meal-based diets is necessary for the optimum growth of 5- to 40-g (Phase-I) and 110- to 240-g (Phase-II) hybrid striped bass (HSB), as well as their intestinal health. Although glycine serves as an essential substrate for syntheses of creatine and glutathione (GSH) in mammals (e.g., pigs), little is known about these metabolic pathways or their nutritional regulation in fish. This study tested the hypothesis that glycine supplementation enhances the activities of creatine- and GSH-forming enzymes as well as creatine and GSH availabilities in tissues of hybrid striped bass (HSB; *Morone saxatilis♀ × Morone chrysops♂*).

**Methods:**

Phase-I and Phase-II HSB were fed a soybean meal-based diet supplemented with 0%, 1%, or 2% glycine for 8 weeks. At the end of the 56-d feeding, tissues (liver, intestine, skeletal muscle, kidneys, and pancreas) were collected for biochemical analyses.

**Results:**

In contrast to terrestrial mammals and birds, creatine synthesis occurred primarily in skeletal muscle from all HSB. The liver was most active in GSH synthesis among the HSB tissues studied. In Phase-I HSB, supplementation with 1% or 2% glycine increased (*P* < 0.05) concentrations of intramuscular creatine (15%–19%) and hepatic GSH (8%–11%), while reducing (*P* < 0.05) hepatic GSH sulfide (GSSG)/GSH ratios by 14%–15%, compared with the 0-glycine group; there were no differences (*P* > 0.05) in these variables between the 1% and 2% glycine groups. In Phase-II HSB, supplementation with 1% and 2% glycine increased (*P* < 0.05) concentrations of creatine and GSH in the muscle (15%–27%) and liver (11%–20%) in a dose-dependent manner, with reduced ratios of hepatic GSSG/GSH in the 1% or 2% glycine group. In all HSB, supplementation with 1% and 2% glycine dose-dependently increased (*P* < 0.05) activities of intramuscular arginine:glycine amidinotransferase (22%–41%) and hepatic γ-glutamylcysteine synthetase (17%–37%), with elevated activities of intramuscular guanidinoacetate methyltransferase and hepatic GSH synthetase and GSH reductase in the 1% or 2% glycine group. Glycine supplementation also increased (*P* < 0.05) concentrations of creatine and activities of its synthetic enzymes in tail kidneys and pancreas, and concentrations of GSH and activities of its synthetic enzymes in the proximal intestine.

**Conclusions:**

Skeletal muscle and liver are the major organs for creatine and GSH syntheses in HSB, respectively. Dietary glycine intake regulates creatine and GSH syntheses by both Phase-I and Phase-II HSB in a tissue-specific manner. Based on the metabolic data, glycine is a conditionally essential amino acid for the growing fish.

**Supplementary Information:**

The online version contains supplementary material available at 10.1186/s40104-024-01024-5.

## Introduction

**Fig. 1:**
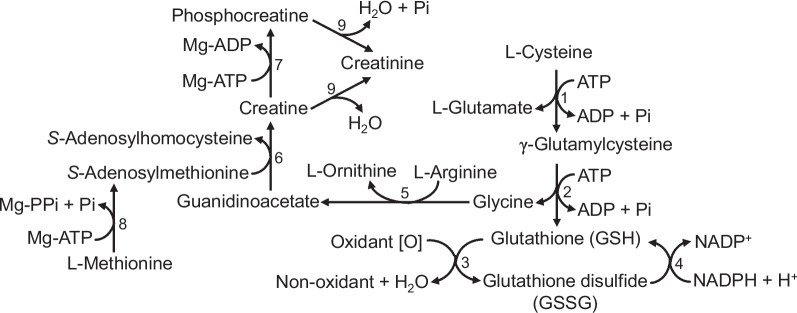
Synthesis of creatine and glutathione in fish. Creatine is produced from glycine in the presence of L-arginine and L-methionine in fish (primarily in skeletal muscle) and is phosphorylated to phosphocreatine to store energy in tissues. In the animals, creatine and phosphocreatine are spontaneously converted into creatinine at 1.7%/d. On the other hand, glutathione is formed from glycine in the presence of L-cysteine and L-glutamate in fish (primarily the liver). In response to oxidants, glutathione is oxidized to glutathione disulfide, which is enzymatically reduced to glutathione in the presence of NADPH + H^+^. The enzymes that catalyze these reactions are: (1) γ-glutamyl-cysteine synthetase; (2) glutathione synthetase; (3) nonenzymatic reaction; (4) glutathione reductase; (5) arginine:glycine amidinotransferase; (6) guanidinoacetate methyltransferase; (7) creatine kinase; (8) *S*-adenosylmethionine synthetase; and (9) non-enzymatic (spontaneous) reaction

Creatine (essential for energy metabolism) and glutathione (GSH; an important non-enzymatic antioxidant) are major metabolites of glycine [the simplest amino acid (AA)] in animals (Fig. 1). Specifically, arginine:glycine amidinotransferase (AGAT) and guanidinoacetate *N*-methyltransferase (GAMT) convert glycine into creatine in the presence of arginine and methionine, whereas γ-glutamylcysteine synthetase and glutathione synthetase catalyze the formation of GSH from glycine, glutamate and cysteine [1]. Thus, a sufficient provision of glycine (the most abundant AA in the body) [2] (e.g., 13.1 and 13.0 g/kg of body weight in juvenile HSB and largemouth bass, respectively [3]) is crucial for supporting energy metabolism and protein synthesis in animals while removing oxidants (including reactive oxygen species) and xenobiotics during rapid periods of their growth [1, 4]. In addition, glycine is used for the formation of serine (participating in one-carbon metabolism), purines (constituents of nucleic acids), and heme (an essential component of hemoglobin, myoglobin, and heme-containing enzymes) with enormous physiological functions in vertebrates including fish [1].

Oxidation of GSH results in the formation of its disulfide (GSSG), with the ratio of GSSG/GSH being a useful indicator of oxidative stress in tissues [5]. Under physiological conditions, a small proportion (1.7%/d) of creatine and phosphocreatine is spontaneously converted into creatinine for excretion [6], whereas GSSG is recycled to GSH by glutathione reductase [1]. Compared with terrestrial mammals (e.g., pigs) and birds (e.g., chickens), the skeletal muscle of fish appears to contain more creatine and phosphocreatine [7]. Through actively storing energy in the form of phosphocreatine via the action of creatine kinase, creatine and phosphocreatine constitute a major energy buffer system in the body. By contrast, in comparison with terrestrial mammals and birds, fish have lower concentrations of GSH in tissues but live in an environment with much higher loads of pathogens (including bacteria, viruses, fungi, and parasites) and therefore have higher risks for oxidative stress and infections [8]. Thus, glycine is particularly important for the nutrition, growth, health, and survival of fish [9].

Hybrid striped bass (HSB) was first produced in South Carolina (USA) in the mid-1960s by fertilizing the eggs of the striped bass (*Morone saxatilis ♀*) with the sperm of the white bass (*M. chrysops ♂*) [10]. The United States is the top producer of HSB in the world as both food and sport fish, with the value of domestic sales ranking #3 after catfish and trout [11]. The HSB has been introduced to many other countries and regions including Mexico, Europe, Asia, and Israel [12]. A major challenge facing the global HSB industry is to reduce the cost of production through the use of plant-sourced protein feedstuffs as an alternative to fishmeal [11]. We recently reported that supplementing 1% or 2% glycine to soybean meal (SBM)-based diets is necessary for the optimum growth of 5- to 40-g (Phase-I) [13] and 110- to 240-g (Phase-II) [14] HSB. It is possible that the formation of GSH and creatine contributes to the beneficial role of glycine in fish growth [15], but currently little is known about these metabolic pathways or their nutritional regulation in aquatic animals. In terrestrial animals, the liver is most active for the production of GSH for transport to other tissues, whereas the synthesis of creatine relies on the interorgan cooperation of AAs involving primarily the kidneys (with a high AGAT activity) and liver (with a high GAMT activity) [1]. There is a suggestion that fish may synthesize creatine predominantly in skeletal muscle based on the presence of high mRNA levels for both AGAT and GAMT in this tissue [16], but data on enzymatic activities are lacking.

In view of the foregoing, this study tested the novel hypothesis that dietary supplementation with glycine to HSB may enhance the activities of creatine- and GSH-synthetic enzymes, as well as creatine and GSH concentrations in tissues. Such work is expected to provide the much-needed basic information about AA metabolism in fish, which can broadly be translated into new dietary strategies to enhance their growth performance and health.

## Materials and methods

This study was approved by The Institutional Animal Care and Use Committee of Texas A&M University (College Station, TX, USA) in accordance with the Animal Welfare Act and Regulations of the United States Department of Agriculture.

### Diets, animals, tissue collection, and chemicals

The fish used for the present research were the cohorts of fish from our published studies involving two phases of feeding [Phase-I (approximately 5–40 g) and Phase-II (approximately 110–240 g)] supplemented with 0%, 1% and 2% glycine [13, 14]. The composition of the diets has been reported previously [13, 14]. The diets contained approximately 44% crude protein and 9.6% lipids on a DM basis that met the National Research Council (NRC)-recommended nutrient requirements for HSB [17]. Briefly, in Phase-I, HSB of similar size (a mean initial body weight of 5.3 g) were fed for 8 weeks a SBM (58%, DM basis)-based diet supplemented with 0%, 1%, or 2% of glycine, with L-alanine serving as the isonitrogenous control. There were four tanks (15 fish/tank) per dietary treatment. The fish were fed their respective diets to apparent satiation twice per day. At the end of the 56-d feeding trial, samples of the liver, proximal intestine, skeletal muscle, and kidneys (head and tail kidneys combined) were collected randomly from two fish/tank, placed immediately in liquid nitrogen, and stored at  −80 °C. Pancreas could not be collected due to its very small size. The Phase-II experiment was conducted in the same manner as Phase-I, except that (a) the mean initial body weight of HSB was approximately 110 g; (b) there were four tanks (4 fish/tank) per dietary treatment; and (c) head kidneys, tail kidneys, and pancreas were also obtained from the fish. In both Phase-I and Phase-II, tissues from fish were not pooled. All measurements were made in individual fish.

For laboratory analyses, all reagents and high-performance liquid chromatography (HPLC) columns were obtained from Sigma Chemicals (St. Louis, MO, USA), unless specified otherwise. HPLC-grade water and methanol were procured from Fisher Scientific (Houston, TX, USA).

### Analyses of GSH and GSSG in tissues

Tissues from individual fish were analyzed for GSH and GSSG by HPLC with a Model 2475 fluorescence detector (Waters Inc., Milford, MA, USSA) according to our established methods, including 20–25 °C for column temperatures and a combined flow rate of 1.1 mL/min [18]. Briefly, a tissue (~ 50 mg) was homogenized in 1.5 mL of a mixture of 1.5 mol/L HClO_4_ and 12 mmol/L iodoacetic acid (v:v = 1:1), and the homogenate was neutralized with 0.375 mL of 2 mol/L K_2_CO_3_. The neutralized solution was analyzed for GSH and GSSG. For the analysis of GSH, a 50-µL sample was mixed with 50 µL of 25 mmol/L iodoacetic acid (an alkylating agent) for 10 min at 25 °C to convert GSH into *S*-carboxymethyl-GSH. The latter was derivatized with *o*-phthaldialdehyde (OPA) to form a highly fluorescent product for detection (excitation wavelength = 340 nm and emission wavelength = 450 nm). For the analysis of GSSG, a 50-µL sample was mixed with 100-µL of 28 mmol/L 2-mercaptoethanol (a reducing agent) for 5 min at 25 °C to convert GSSG to GSH, and the latter were analyzed as described above. Total glutathione (reduced plus oxidized forms) was calculated as the sum of GSH plus ½ GSSG. GSG and GSSG standards were used for the quantification of these substances in samples.

### Analyses of creatine and related metabolites in plasma and tissues

Plasma and tissues were analyzed for creatine, phosphocreatine, creatinine, and guanidinoacetate by HPLC as we described previously [19]. These assays were based on our observation that, in an alkaline solution, creatine (an open-chain), but not creatinine (a cyclic structure), phosphocreatine, or guanidinoacetate, rapidly reacted with benzoin to yield a fluorescent derivative. Boiling converts creatinine and guanidinoacetate into creatine and glycocyamidine, respectively, for subsequent derivatization with benzoin, whereas creatine kinase dephosphorylates phosphocreatine to creatine. Briefly, plasma (100 µL) was mixed with an equal volume of 1.5 mol/L HClO_4_, followed by the addition of 50 µL of 2 mol/L K_2_CO_2_. In addition, 50 mg of frozen tissue was homogenized in 1 mL of 1.5 mol/L HClO_4_, followed by the addition of 0.5 mL of 2 mol/L K_2_CO_2_. The neutralized solution was centrifuged at 10,000 × *g* for 1 min to obtain the supernatant fluid for analysis. For the determination of creatine, 20 µL of a sample (or creatine standard) plus 80 µL of HPLC-grade water (not subject to boiling) was programed to mix with 30 mmol/L benzoin (5 µL), 100 mmol/L β-mercaptoethanol plus 200 mmol/L sodium sulfite (5 µL), and 2 mol/L KOH (10 µL) in an autosampler. The creatine-benzoin derivative was then separated on a Supelco C18 column by Solvent A (0.1 mol/L sodium acetate) and Solvent B (100% HPLC-grade methanol). Detection was at an excitation wavelength of 325 nm and an emission wavelength of 425 nm. For the measurement of creatinine, a sample was boiled for 15 min to convert creatinine into creatine, which was determined as described previously. The difference in creatine between boiling (creatine + creatinine) and no boiling (creatine) was calculated as creatinine. The boiled sample was analyzed for the analysis of guanidinoacetate after its derivatization with benzoin, as described above. For the analysis of phosphocreatine, 50 µL sample was incubated at 37 °C for 30 min with 10 µL of creatine kinase (1 mg/mL), and 50 µL of 10 mmol/L ADP plus 100 mmol/L MgCl_2_ to convert phosphocreatine into creatine. The limits of detection for creatine, creatinine, phosphocreatine, and guanidinoacetate in biological samples were 0.1, 0.1, 0.5, and 0.1 µmol/L, respectively.

### Analysis of activities of enzymes for creatine synthesis in tissues

Tissue (~ 0.1 g) was homogenized in 0.5 mL of sodium phosphate buffer (pH 7.5) that contained 4 mmol/L dithiothreitol (DTT) and 0.1% stock solution of protease inhibitors [a mixture of aprotinin, chymostatin, pepstatin A, and phenylmethylsulfonylfluoride (2.5 µg/mL) in dimethyl sulfoxide], followed by two cycles of freezing (−80 °C) and thawing (25 °C). The whole homogenate was stored at −80 °C to determine the activities of AGAT and GAMT, as described by Verhoeven et al. [20] and Carducci et al. [21], except that the temperature of the assay solution was 26 °C (the same as the water tank for fish housing) and that guanidinoacetate, creatine, and creatine phosphate were analyzed using HPLC methods [19].

#### Determination of AGAT activity

Tissue homogenate (100 µL) was incubated with 100 µL of 30 mmol/L L-arginine/glycine for 30 min at 26 °C. At the end of the incubation, the reaction was stopped by the addition of 100 µL of 1.5 mol/L HClO_4_. After 2 min, 50 µL of 2 mol/L K_2_CO_3_ was added to the acidified assay mixture. The neutralized solution was centrifuged at 10,000 × *g* for 1 min, and the supernatant fluid was stored at −80 °C for the analysis of guanidinoacetate. Blanks, in which 100 µL of 1.5 mol/L HClO_4_ was added to assay tubes before tissue homogenate, were included. AGAT activity was linear with the time of incubation and the amount of enzyme extract in the assay solution.

#### Determination of GAMT activity

Tissue homogenate was centrifuged at 10,000 × *g* for 2 min. The supernatant fluid (100 µL) was incubated with 100 µL of 2 mmol/L guanidinoacetate/*S*-adenosylmethionine for 30 min at 26 °C. At the end of the incubation, the reaction was terminated by the addition of 100 µL of 1.5 mol/L HClO_4_. After 2 min, 50 µL of 2 mol/L K_2_CO_3_ was added to the acidified assay mixture. The neutralized solution was centrifuged at 10,000 × *g* for 1 min, and the supernatant fluid was stored at −80 °C for the analysis of creatine, as described previously. Blanks, in which 100 µL of 1.5 mol/L HClO_4_ was added to each assay tube before tissue homogenate, were included. GAMT activity was linear with the time of incubation and the amount of enzyme extract in the assay solution.

### Determination of activities of enzymes for GSH formation in tissues

#### Determination of activities of GSH-forming enzymes

Tissue samples (approximately 0.25 g) were homogenized with 2 mL of 100 mmol/L Tris buffer (pH 8.0) containing 2 mmol/L EDTA, 3 mmol/L dithiothreitol (DTT), 0.5% Triton X-100, and 0.1% protease inhibitors (aprotinin, chymostatin, pepstatin A, and phenylmethylsulfonylfluoride, 2.5 μg/mL each in dimethyl sulfoxide). The homogenates were centrifuged at 10,000 × *g* for 1 min at 25 °C. The supernatant fluid (enzyme extract) was used for assays of enzymatic activities.

#### γ-Glutamylcysteine synthetase

The enzymatic activity of γ-glutamylcysteine synthetase was measured as described by Nardi and Cipollaro [22], with modifications. Briefly, enzyme extract (40 µL) was incubated with 80 µL of 100 mmol/L Tris buffer (pH 8.0), 40 µL of 100 mmol/L MgCl_2_/30 mmol/L ATP/10 mmol/L EDTA/250 mmol/L KCl, and 40 µL of 30 mmol/L DTT/25 mmol/L cysteine/75 mmol/L L-glutamate for 15 min at 26 °C. At the end of the incubation, 50 µL of 1.5 mol/L HClO_4_ was added to the incubation tube to terminate the reaction. After 2-min standing at 25 °C, 25 µL of 2 mol/L K_2_CO_3_ was added to neutralize the solution. For blank tubes, the assay solution contained all the components as those in the sample tubes except that 50 µL of 1.5 mol/L HClO_4_ was added to the assay solution before the inclusion of enzyme extract. Finally, all the tubes were centrifuged at 10,000 × *g* for 1 min, and the supernatant fluid was used for the HPLC analysis of γ-glutamylcysteine through the precolumn derivatization with OPA as described above, except that the solvent gradients were: 0–1 min: 97% Solvent A (0.1 mol/L sodium acetate) and Solvent B (HPLC-graded methanol); 1.1–6.5 min: 86% solvent A and 14% solvent B; 6.6–9.0 min: 100% solvent B; and 9.1–16 min: 97% solvent A and 3% solvent B.

#### Glutathione synthetase

The enzymatic activity of GSH synthetase was measured over a 15-min period, as described by Nardi and Cipollaro [22]. The solution for the assay of GSH synthetase activity was the same as that for γ-glutamylcysteine synthetase above, except that the cysteine and L-glutamate components were replaced with 25 mmol/L γ-glutamylcysteine and 150 mmol/L glycine. The temperature of the incubation of the assay mixture was 26 °C. After the reaction was terminated, the assay solution was used for the HPLC analysis of GSH and GSSG, as described above.

#### Glutathione reductase

The enzymatic activity of GSH reductase (the enzyme converting GSSG back to GSH) was measured as described by Goldberg and Spooner [23], except that a 96-well microplate reader was used. Briefly, enzyme extract (10 µL) was mixed with 160 µL of 125 mmol/L potassium phosphate buffer (pH 7.2), 10 µL of 10 mmol/L EDTA/0.1 mmol/L FAD, and 10 µL of 44 mmol/L GSSG. After 5-min standing at 25 °C, 10 µL of 3.4 mmol/L NADPH was added to each sample well, followed by the reading of absorbance at 340 nm for 0, 5, 10, and 15 min. Blanks were included by replacing 3.4 mmol/L NADPH with 125 mmol/L potassium phosphate buffer (pH 7.2). The molar extinction coefficient of NADPH (6.22 × 10^3^•mol/L^−1^•cm^−1^) was used to calculate the disappearance of NADPH due to the conversion of GSSG into GSH.

Activities of all these GSH-forming enzymes were linear with the time of incubation and the amount of tissue extract in the assay solutions.

### Statistical analyses

Results are expressed as mean ± SEM. Data were first tested for normality using the Shapiro–Wilk Test and their normal distribution was confirmed (*P* > 0.05). Analyses of data on metabolite concentrations and enzyme activities were performed using the JMP 15 Pro software (Cary, NC, USA) for one-way analysis of variance. Differences among treatment means were determined using the Student–Newman–Keuls (SNK) multiple comparison test. This method was chosen because it is more stringent than the Duncan test and more powerful than the Tukey method to detect significant differences among the means of treatment groups [24]. Thus, the SNK procedure is a good compromise between power and conservatism and is commonly used in biological research [24]. A probability value of *P* ≤ 0.05 was taken to indicate statistical significance.

## Results

### Phase-I HSB

#### Effects of dietary glycine supplementation on concentrations of creatine and its metabolites in tissues

Data on the concentrations of creatine and its metabolites in the tissues of Phase-I HSB are summarized in Table 1. Dietary supplementation with 2% glycine increased (*P* < 0.05) concentrations of guanidinoacetate, creatine, phosphocreatine, and creatine plus phosphocreatine in the liver when compared with the 0% glycine group. In fish fed the 1% glycine diet, concentrations of creatine and creatine plus phosphocreatine were elevated (*P* < 0.05) in the liver, as compared with the 0% glycine group. Hepatic concentrations of guanidinoacetate, creatine, phosphocreatine, or creatine plus phosphocreatine did not differ (*P* > 0.05) between the 1% and 2% glycine groups. Creatinine concentrations in this tissue did not differ (*P* > 0.05) among the three groups of fish.

**Table 1:** Concentrations of creatine and its metabolites in the tissues of hybrid striped bass^1^

Tissue	Treatment	Creatinine^2^	GAA^3^	Creatine^2^	CrP^2^	Cr + CrP^2^
Phase-I hybrid striped bass						
Liver	0% Gly	0.653 ± 0.009	4.07 ± 0.34^b^	0.295 ± 0.011^b^	0.428 ± 0.019	0.722 ± 0.020^b^
	1% Gly	0.666 ± 0.008	4.94 ± 0.36^a,b^	0.364 ± 0.014^a^	0.455 ± 0.026	0.819 ± 0.037^a^
	2% Gly	0.660 ± 0.003	5.69 ± 0.39^a^	0.414 ± 0.031^a^	0.465 ± 0.038	0.878 ± 0.041^a^
	*P*-value	0.452	0.017	0.002	0.648	0.013
Kidney	0% Gly	1.27 ± 0.022	30.7 ± 2.0^c^	2.59 ± 0.05^c^	4.46 ± 0.27	7.05 ± 0.27^b^
	1% Gly	1.27 ± 0.024	48.8 ± 2.2^b^	3.03 ± 0.08^b^	4.64 ± 0.35	7.67 ± 0.29^b^
	2% Gly	1.31 ± 0.038	76.6 ± 4.6^a^	3.71 ± 0.13^a^	4.87 ± 0.29	8.58 ± 0.30^a^
	*P*-value	0.538	< 0.001	< 0.001	0.642	0.004
Skeletal muscle	0% Gly	11.3 ± 0.40	61.5 ± 3.6^c^	32.0 ± 0.34^b^	58.3 ± 1.9^b^	90.2 ± 2.0^b^
	1% Gly	11.4 ± 0.33	73.4 ± 1.4^b^	36.9 ± 1.0^a^	75.1 ± 2.5^a^	112 ± 2.2^a^
	2% Gly	10.8 ± 0.37	86.3 ± 5.8^a^	38.8 ± 1.6^a^	77.1 ± 2.5^a^	116 ± 3.1^a^
	*P*-value	0.478	0.001	< 0.001	< 0.001	< 0.001
Proximal intestine	0% Gly	2.07 ± 0.026	37.2 ± 1.8^b^	2.12 ± 0.04	4.57 ± 0.20	6.69 ± 0.20
	1% Gly	1.99 ± 0.028	39.9 ± 0.75^a,b^	2.11 ± 0.03	4.79 ± 0.33	6.90 ± 0.35
	2% Gly	2.03 ± 0.036	42.5 ± 1.1^a^	2.22 ± 0.06	5.07 ± 0.42	7.29 ± 0.44
	*P*-value	0.200	0.029	0.187	0.569	0.471
Plasma	0% Gly	0.053 ± 0.002	0.021 ± 0.001^c^	0.214 ± 0.007^b^	ND	0.214 ± 0.007^b^
	1% Gly	0.051 ± 0.002	0.025 ± 0.001^b^	0.244 ± 0.006^a^	ND	0.244 ± 0.006^a^
	2% Gly	0.051 ± 0.003	0.029 ± 0.001^a^	0.247 ± 0.005^a^	ND	0.247 ± 0.005^a^
	*P*-value	0.798	< 0.001	0.001	−	0.001
Phase-II hybrid striped bass						
Liver	0% Gly	0.705 ± 0.026	ND	0.400 ± 0.039	0.517 ± 0.030	0.917 ± 0.027
	1% Gly	0.700 ± 0.032	ND	0.437 ± 0.042	0.569 ± 0.031	1.01 ± 0.042
	2% Gly	0.680 ± 0.006	ND	0.461 ± 0.040	0.585 ± 0.028	1.05 ± 0.044
	*P*-value	0.742	−	0.569	0.261	0.063
Head kidney	0% Gly	1.82 ± 0.015	ND	1.97 ± 0.06	3.88 ± 0.35	5.85 ± 0.386
	1% Gly	1.82 ± 0.025	ND	1.94 ± 0.12	3.95 ± 0.22	5.89 ± 0.311
	2% Gly	1.81 ± 0.021	ND	1.95 ± 0.06	4.21 ± 0.20	6.16 ± 0.219
	*P*-value	0.926	−	0.970	0.661	0.751
Tail kidney	0% Gly	0.266 ± 0.020	22.8 ± 2.8	3.60 ± 0.20	4.10 ± 0.20^b^	7.67 ± 0.33^b^
	1% Gly	0.255 ± 0.018	24.5 ± 3.4	3.61 ± 0.20	5.15 ± 0.47^a^	8.76 ± 0.57^a,b^
	2% Gly	0.289 ± 0.016	26.7 ± 2.8	3.82 ± 0.15	5.91 ± 0.30^a^	9.73 ± 0.43^a^
	*P*-value	0.414	0.661	0.647	0.005	0.015
Skeletal muscle	0% Gly	8.37 ± 0.28	507 ± 50	36.2 ± 0.84^c^	67.0 ± 2.3^c^	103 ± 3.0^c^
	1% Gly	8.39 ± 0.24	511 ± 35	41.4 ± 1.4^b^	82.0 ± 3.8^b^	123 ± 5.0^b^
	2% Gly	8.15 ± 0.31	542 ± 37	45.8 ± 1.5^a^	93.2 ± 4.9^a^	139 ± 6.2^a^
	*P*-value	0.797	0.810	< 0.001	< 0.001	< 0.001
Pancreas	0% Gly	0.726 ± 0.010	11.4 ± 1.1^b^	0.80 ± 0.04^c^	1.05 ± 0.11^c^	1.84 ± 0.12^c^
	1% Gly	0.720 ± 0.013	14.2 ± 1.0^a,b^	1.04 ± 0.04^b^	1.75 ± 0.14^b^	2.79 ± 0.11^b^
	2% Gly	0.732 ± 0.016	16.2 ± 1.5^a^	1.43 ± 0.05^a^	2.20 ± 0.12^a^	3.63 ± 0.16^a^
	*P*-value	0.816	0.033	< 0.001	< 0.001	< 0.001
Proximal intestine	0% Gly	2.37 ± 0.027	35.4 ± 2.6	2.96 ± 0.14	3.71 ± 0.77	6.67 ± 0.65
	1% Gly	2.20 ± 0.089	31.0 ± 2.6	2.90 ± 0.25	4.38 ± 0.70	7.27 ± 0.85
	2% Gly	2.35 ± 0.048	37.5 ± 2.3	2.75 ± 0.11	4.86 ± 0.75	7.61 ± 0.67
	*P*-value	0.126	0.198	0.693	0.554	0.658
Plasma	0% Gly	0.058 ± 0.017	0.035 ± 0.001^c^	0.240 ± 0.011^b^	ND	0.240 ± 0.013^b^
	1% Gly	0.057 ± 0.018	0.040 ± 0.002^b^	0.277 ± 0.011^a^	ND	0.277 ± 0.011^a^
	2% Gly	0.058 ± 0.009	0.046 ± 0.001^a^	0.285 ± 0.009^a^	ND	0.285 ± 0.009^a^
	*P*-value	0.999	< 0.001	0.020	−	0.020

*Cr* Creatine, *GAA* Guanidinoacetate, *CrP* phosphocreatine, *ND* Not detected

^1^Values are mean ± SEM, *n* = 8 fish per treatment group. Fish received dietary supplementation with 0%, 1%, or 2% glycine for 56 d. L-Alanine was used as the isonitrogenous control. Tissues were obtained from the fish at the end of the study. Plasma was obtained from the blood through centrifugation

^2^Data are expressed as µmol/g of fresh tissues or µmol/mL of plasma

^3^Data are expressed as nmol/g of fresh tissues or nmol/mL of plasma

^a−c^Within a column, means not sharing the same superscript letter differ (*P* < 0.05)

Dietary supplementation with 1% and 2% glycine to Phase-I HSB increased concentrations of guanidinoacetate and creatine in the kidneys in a dose-dependent manner (Table 1). Specifically, when compared with the 0% glycine group, supplementation with 1% and 2% glycine increased (*P* < 0.05) renal concentrations of guanidinoacetate by 59% and 150%, respectively and of creatine by 17% and 43%, respectively. When compared with the 0% glycine group, only 2% supplemental glycine enhanced (*P* < 0.05) creatine plus phosphocreatine concentrations in this tissue. Creatinine concentrations in the kidneys did not differ (*P* > 0.05) among all treatment groups.

Dietary supplementation with 1% and 2% glycine to Phase-I HSB increased guanidinoacetate concentrations in skeletal muscle in a dose-dependent manner (Table 1). Specifically, 1% and 2% supplemental glycine increased (*P* < 0.05) guanidinoacetate concentrations in this tissue by 14% and 40%, respectively, compared with the 0% glycine group. When compared with the 0% glycine group, 1% and 2% supplemental glycine increased (*P* < 0.05) concentrations of creatine, phosphocreatine, and creatine plus phosphocreatine in skeletal muscle by 15%, 29%, and 24%, respectively. Creatinine concentrations in the kidneys did not differ (*P* > 0.05) among all treatment groups. In contrast, guanidinoacetate concentrations in the proximal intestine were elevated (*P* < 0.05) in Phase-I HSB fed the 2% glycine diet, compared with the 0% glycine group (Table 1). Concentrations of creatine and creatine phosphate in this tissue were not affected (*P* > 0.05) by dietary supplementation with either 1% or 2% glycine.

Concentrations of guanidinoacetate and creatine in the plasma responded to dietary glycine supplementation to Phase-I HSB in a different manner than those in the tissues. Compared with the 0% glycine group, dietary supplementation with 1% and 2% glycine dose-dependently increased (*P* < 0.05) concentrations of guanidinoacetate in the plasma of HSB by 20% and 37%, respectively. In contrast, creatine concentrations in plasma did not differ (*P* > 0.05) between the 1% and 2% groups, but values were 14% greater (*P* < 0.05) than those in the 0% glycine group. No difference (*P* > 0.05) was detected in plasma creatinine concentrations among the three groups of fish.

#### Effects of dietary glycine supplementation on concentrations of GSH and GSSG in tissues

Data on the concentrations of GSH and GSSG in the tissues of Phase-I HSB are summarized in Table 2. When compared with the 0 glycine group, dietary supplementation with 1% and 2% glycine increased (*P* < 0.05) concentrations of GSH and total glutathione in the liver to a similar extent (i.e., by 8% and 6%, respectively). The ratios of GSSG/GSH (mol/mol) in the liver were reduced (*P* < 0.05) by 14% by dietary supplementation with 1% and 2% glycine. No difference (*P* > 0.05) in hepatic GSSG concentrations was observed among all treatment groups.

**Table 2:** Concentrations of glutathione and glutathione disulfide in the tissues of hybrid striped bass^1^

Tissue	Variable	0% Glycine	1% Glycine	2% Glycine	*P*-value
Phase-I hybrid striped bass					
Liver	GSH	1,717 ± 32^b^	1,857 ± 26^a^	1,904 ± 16^a^	< 0.001
	GSSG	156 ± 6.7	145 ± 9.5	146 ± 10	0.630
	GSSG:GSH	0.091 ± 0.005^a^	0.078 ± 0.003^b^	0.077 ± 0.003^b^	0.028
	Total glutathione^†^	2,028 ± 30^b^	2,146 ± 37^a^	2,197 ± 26^a^	0.003
Kidney	GSH	326 ± 7.4	337 ± 11	340 ± 4.9	0.456
	GSSG	30.1 ± 1.1^a^	26.5 ± 0.85^b^	27.1 ± 0.85^b^	0.029
	GSSG:GSH	0.092 ± 0.002^a^	0.079 ± 0.002^b^	0.080 ± 0.002^b^	< 0.001
	Total glutathione^†^	386 ± 9.2	390 ± 12	394 ± 6.1	0.836
Skeletal muscle	GSH	190 ± 3.5	194 ± 2.0	198 ± 1.6	0.839
	GSSG	15.8 ± 0.65^a^	14.0 ± 0.58^b^	13.3 ± 0.63^b^	0.027
	GSSG:GSH	0.083 ± 0.004^a^	0.072 ± 0.003^b^	0.067 ± 0.003^b^	0.009
	Total glutathione^†^	222 ± 3.7	222 ± 2.3	224 ± 2.1	0.844
Proximal intestine	GSH	490 ± 11^c^	621 ± 24^b^	703 ± 17^a^	< 0.001
	GSSG	44.0 ± 0.74^c^	49.1 ± 2.2^b^	55.9 ± 2.0^a^	< 0.001
	GSSG:GSH	0.090 ± 0.002^a^	0.079 ± 0.002^b^	0.080 ± 0.003^b^	0.006
	Total glutathione^†^	578 ± 12^c^	719 ± 27^b^	814 ± 20^a^	< 0.001
Phase-II hybrid striped bass					
Liver	GSH	1,516 ± 53^c^	1,678 ± 20^b^	1,814 ± 41^a^	< 0.001
	GSSG	145 ± 4.9	145 ± 2.9	149 ± 4.8	0.752
	GSSG:GSH	0.096 ± 0.002^a^	0.086 ± 0.001^b^	0.083 ± 0.003^b^	< 0.001
	Total glutathione^†^	1,806 ± 61^c^	1,968 ± 25^b^	2,113 ± 39^a^	< 0.001
Head kidney	GSH	397 ± 21	419 ± 20	427 ± 14	0.509
	GSSG	37.2 ± 2.1	35.2 ± 1.2	35.2 ± 1.5	0.614
	GSSG:GSH	0.094 ± 0.001^a^	0.085 ± 0.003^b^	0.083 ± 0.003^b^	0.013
	Total glutathione^†^	472 ± 25	489 ± 21	497 ± 16	0.695
Tail kidney	GSH	161 ± 4.4	166 ± 4.1	166 ± 5.4	0.687
	GSSG	15.4 ± 0.65	14.2 ± 0.58	13.5 ± 0.60	0.108
	GSSG:GSH	0.095 ± 0.003^a^	0.085 ± 0.002^b^	0.082 ± 0.004^b^	< 0.001
	Total glutathione^†^	192 ± 5.4	195 ± 5.0	193 ± 5.8	0.924
Skeletal muscle	GSH	155 ± 6.2	159 ± 5.7	161 ± 3.9	0.726
	GSSG	14.2 ± 0.62	12.8 ± 0.60	12.7 ± 0.70	0.115
	GSSG:GSH	0.092 ± 0.002^a^	0.080 ± 0.002^b^	0.079 ± 0.004^b^	0.006
	Total glutathione^†^	184 ± 7.2	184 ± 6.8	186 ± 4.8	0.968
Pancreas	GSH	8.62 ± 0.41	8.85 ± 0.23	8.79 ± 0.38	0.890
	GSSG	0.784 ± 0.027	0.802 ± 0.033	0.801 ± 0.036	0.907
	GSSG:GSH	0.092 ± 0.003	0.091 ± 0.003	0.092 ± 0.004	0.971
	Total glutathione^†^	10.2 ± 0.45	10.4 ± 0.28	10.4 ± 0.41	0.915
Proximal intestine	GSH	467 ± 10^c^	541 ± 12^b^	637 ± 19^a^	< 0.001
	GSSG	38.4 ± 1.2	40.6 ± 1.6	42.2 ± 1.5	0.199
	GSSG:GSH	0.085 ± 0.003^a^	0.075 ± 0.003^b^	0.067 ± 0.004^b^	0.004
	Total glutathione^†^	531 ± 7.9^c^	622 ± 14^b^	721 ± 18^a^	< 0.001

*GSH* Glutathione, *GSSG* Glutathione disulfide

^1^Values are expressed as nmol/g of fresh tissue for GSH, GSSG and total glutathione or as mol/mol for GSSG/GSH ratios. Data are mean ± SEM, *n* = 8 fish per treatment group. Fish received dietary supplementation with 0%, 1%, or 2% glycine for 56 d. L-Alanine was used as the isonitrogenous control. Tissues were obtained from the fish at the end of the study

^†^GSH + ½ GSSG

^a–c^Within a row, means not sharing the same superscript letter differ (*P* < 0.05)

GSSG concentrations and GSSG/GSH ratios in the kidneys of Phase-I HSB were reduced (*P* < 0.05) by 12% and 14% in HSB receiving dietary supplementation with 1% and 2% glycine, respectively (Table 2). No change (*P* > 0.05) in concentrations of GSH or total glutathione in the kidneys was detected among all treatment groups. GSSG concentration and GSSG/GSH ratios in the skeletal muscle of Phase-I HSB were reduced (*P* < 0.05) by 11% and 13% in HSB receiving dietary supplementation with 1% and 2% glycine, respectively (Table 2). No change (*P* > 0.05) in concentrations of GSH or total glutathione in the muscle was detected among all treatment groups (Table 2).

Augmenting dietary supplementation with glycine from 0 to 2% increased (*P* < 0.05) concentrations of GSH, GSSG, and total glutathione in the proximal intestine in a dose-dependent manner (Table 2). Specifically, intestinal concentrations of GSH were increased (*P* < 0.05) by 27% and 43% in the 1% and 2% glycine groups, respectively, compared with the 0% glycine group. Intestinal concentrations of GSSG were increased (*P* < 0.05) by 12% and 27% in HSB receiving dietary supplementation with 1% and 2% glycine, respectively. Intestinal concentrations of total glutathione were increased (*P* < 0.05) by 24% and 41% in HSB receiving dietary supplementation with 1% and 2% glycine, respectively. Dietary supplementation with 1% and 2% glycine reduced (*P* < 0.05) the ratio of GSSG/GSH in the proximal intestine by 12%, compared with the 0% glycine group.

#### Effects of dietary glycine supplementation on the activities of enzymes for creatine synthesis in tissues

Data on the activities of enzymes for creatine synthesis in tissues of Phase-I HSB are summarized in Table 3. Dietary glycine supplements increased (*P* < 0.05) AGAT activity in the kidneys and skeletal muscle in a dose-dependent manner. Specifically, 1% and 2% supplemental glycine increased (*P* < 0.05) AGAT activities by 18% and 27% in the kidneys, respectively, and by 25% and 39%, in skeletal muscle, respectively. Glycine supplementation did not change (*P* > 0.05) AGAT activities in the liver and proximal intestine.

**Table 3:** Activities of enzymes for creatine synthesis in the tissues of hybrid striped bass^1^

Enzyme	Tissue	0% glycine	1% Glycine	2% Glycine	*P*-value
Phase-I hybrid striped bass					
AGAT	Liver	1,870 ± 79	1,954 ± 81	1,966 ± 61	0.616
	Kidney	3,030 ± 80^c^	3,572 ± 95^b^	3,843 ± 95^a^	< 0.001
	Skeletal muscle	5,164 ± 202^c^	6,431 ± 310^b^	7,170 ± 224^a^	< 0.001
	Proximal intestine	319 ± 16	326 ± 19	329 ± 15	0.911
GAMT	Liver	2,107 ± 71^b^	2,225 ± 57^ab^	2,359 ± 67^a^	0.041
	Kidney	3,052 ± 88	2,993 ± 143	3,036 ± 144	0.945
	Skeletal muscle	7,234 ± 206^b^	8,875 ± 226^a^	9,402 ± 285^a^	< 0.001
	Proximal intestine	649 ± 30	659 ± 32	669 ± 19	0.878
Phase-II hybrid striped bass					
AGAT	Liver	1,880 ± 89	2,041 ± 79	2,071 ± 106	0.308
	Head kidney	1,941 ± 73	1,935 ± 90	1,904 ± 55	0.931
	Tail kidney	3,509 ± 101^c^	4,361 ± 186^b^	5,086 ± 180^a^	< 0.001
	Skeletal muscle	5,941 ± 161^c^	7,222 ± 181^b^	8,387 ± 105^a^	< 0.001
	Pancreas	3,643 ± 172^b^	4,728 ± 129^a^	4,727 ± 126^a^	< 0.001
	Proximal intestine	261 ± 13	269 ± 12	286 ± 18	0.477
GAMT	Liver	2,066 ± 98^b^	2,240 ± 128^a,b^	2,482 ± 103^a^	0.046
	Head kidney	ND	ND	ND	−
	Tail kidney	4,030 ± 86	4,109 ± 159	4,043 ± 283	0.954
	Skeletal muscle	8,159 ± 351^b^	9,640 ± 387^a^	10,517 ± 204^a^	< 0.001
	Pancreas	5,885 ± 207	5,910 ± 161	5,909 ± 157	0.994
	Proximal intestine	ND	ND	ND	−

*AGAT* Arginine:glycine amidinotransferase, *GAMT* Guanidinoacetate methyltransferase, *ND* Not detected

^1^Values, expressed as nmol/g of fresh tissue/30 min, are mean ± SEM, *n* = 8 fish per treatment group. Fish received dietary supplementation with 0%, 1%, or 2% glycine for 56 d. L-Alanine was used as the isonitrogenous control. Tissues were obtained from the fish at the end of the study

^a−c^Within a row, means not sharing the same superscript letter differ (*P* < 0.05)

When compared with the 0% glycine group, dietary supplementation with 2% glycine Phase-I HSB increased (*P* < 0.05) hepatic GAMT activity by 12%, but dietary supplementation with 1% glycine had no effect (*P* > 0.05) (Table 3). Dietary supplementation with 1% and 2% glycine increased (*P* < 0.05) GAMT activity in skeletal muscle by 23% but had no effect (*P* > 0.05) on GAMT activities in the kidneys or the proximal intestine.

#### Effects of dietary glycine supplementation on the activities of GSH-forming enzymes in tissues

Data on the activities of GSH-forming enzymes in the liver and proximal intestine of Phase-I HSB are summarized in Table 4. In the liver of HSB, dietary supplementation with glycine increased (*P* < 0.05) the activities of γ-glutamylcysteine synthetase and GSH synthetase in a dose-dependent manner. Specifically, when compared with the 0% glycine group, hepatic γ-glutamylcysteine synthetase activities in the 1% and 2% glycine groups were elevated (*P* > 0.05) by 17% and 36%, respectively. Dietary supplementation with 1% and 2% glycine increased (*P* > 0.05) hepatic GSH synthetase activity by 15% and 24%, respectively, compared with the 0% glycine group. Hepatic GSH reductase activity did not differ (*P* > 0.05) between the 0 and 1% glycine groups, but the value was 13% lower (*P* < 0.05) than that in the 2% glycine group. Activities of all these three enzymes in the liver were greater (*P* < 0.05) in the 2% glycine group, compared with the 1% glycine group.

**Table 4:** Activities of enzymes for glutathione formation in the tissues of hybrid striped bass^1^

Enzyme	Tissue	0% Glycine	1% Glycine	2% Glycine	*P*-value
Phase-I hybrid striped bass					
GCS	Liver	101 ± 1.1^c^	118 ± 4.2^b^	137 ± 3.4^a^	< 0.001
	Kidney	10.0 ± 0.87	10.1 ± 0.48	10.3 ± 0.63	0.951
	Skeletal muscle	24.5 ± 0.44	25.0 ± 0.38	24.8 ± 1.3	0.911
	Proximal intestine	108 ± 5.2^b^	124 ± 2.6^a^	132 ± 4.3^a^	0.002
GSHS	Liver	658 ± 17^c^	754 ± 6.4^b^	818 ± 27^a^	< 0.001
	Kidney	396 ± 24	402 ± 19	407 ± 21	0.936
	Skeletal muscle	222 ± 3.1	229 ± 13	228 ± 9.2	0.850
	Proximal intestine	134 ± 6.0^b^	148 ± 4.7^a^	150 ± 1.9^a^	0.042
GSHR	Liver	5,850 ± 95^b^	5,934 ± 236^b^	6,793 ± 281^a^	0.011
	Kidney	1,404 ± 88^b^	1,712 ± 72^a^	1,768 ± 102^a^	0.018
	Skeletal muscle	1,086 ± 63^b^	1,324 ± 71^a^	1,350 ± 65^a^	0.019
	Proximal intestine	7,577 ± 115^b^	8,259 ± 140^a^	8,425 ± 161^a^	< 0.001
Phase-II hybrid striped bass					
GCS	Liver	108 ± 2.6^c^	127 ± 3.2^b^	148 ± 4.3^a^	< 0.001
	Head kidney	6.22 ± 0.46	6.10 ± 0.32	6.18 ± 0.23	0.970
	Tail kidney	14.8 ± 1.2	14.6 ± 0.75	14.7 ± 0.67	0.988
	Skeletal muscle	19.7 ± 0.23	20.0 ± 0.61	20.0 ± 1.3	0.958
	Pancreas	25.3 ± 0.72	26.4 ± 0.66	25.6 ± 1.1	0.645
	Proximal intestine	104 ± 5.5^b^	122 ± 3.0^a^	126 ± 2.6^a^	0.002
GSHS	Liver	579 ± 11^b^	740 ± 4.6^a^	751 ± 8.3^a^	< 0.001
	Head kidney	684 ± 23	691 ± 17	682 ± 4.2	0.923
	Tail kidney	339 ± 21	340 ± 10	337 ± 14	0.991
	Skeletal muscle	194 ± 8.7	196 ± 6.9	203 ± 9.2	0.728
	Pancreas	294 ± 8.2	299 ± 16	301 ± 14	0.928
	Proximal intestine	162 ± 6.0^c^	189 ± 8.7^b^	209 ± 5.2^a^	< 0.001
GSHR	Liver	4,617 ± 225^b^	4,726 ± 53^b^	5,246 ± 119^a^	0.017
	Head kidney	1,007 ± 73^b^	1,390 ± 92^a^	1,387 ± 87^a^	0.005
	Tail kidney	1,481 ± 66^b^	1,788 ± 68^a^	1,800 ± 63^a^	0.003
	Skeletal muscle	775 ± 48^b^	1,125 ± 77^a^	1,164 ± 63^a^	< 0.001
	Pancreas	1,791 ± 71	1,752 ± 87	1,799 ± 61	0.891
	Proximal intestine	9,137 ± 168^b^	10,302 ± 269^a^	10,386 ± 231^a^	0.001

*GCS* γ-glutamylcysteine synthetase, *GSHR* glutathione reductase, *GSHS* glutathione synthetase

^1^Values, expressed as nmol/g tissue/15 min, are mean ± SEM, *n* = 8 fish per treatment group. Fish received dietary supplementation with 0%, 1%, or 2% glycine for 56 d. L-Alanine was used as the isonitrogenous control. Tissues were obtained from the fish at the end of the study

^a−c^Within a row, means not sharing the same superscript letter differ (*P* < 0.05)

In the proximal intestine of Phase-I HSB, activities of GSH reductase, γ-glutamylcysteine synthetase, and GSH synthetase were 8% and 10% lower (*P* < 0.05) in the 0% glycine group than those in the 1% and 2% glycine groups, respectively (Table 4). Intestinal activities of all these three enzymes did not differ (*P* > 0.05) between the 1% and 2% glycine groups. In the kidneys and skeletal muscle, both 1% and 2% glycine supplementations to Phase-I HSB had the same effect in increasing (+ 22%; *P* < 0.05) the activity of GSH reductase (Table 4). Dietary glycine supplementation did not influence (*P* > 0.05) the activities of GSH-forming enzymes in these tissues.

### Phase-II HSB

#### Effects of dietary glycine supplementation on concentrations of creatine and its metabolites in plasma and tissues

Data on the concentrations of creatine and its metabolites in the tissues of Phase-II HSB are summarized in Table 1. Dietary supplementation with glycine did not affect (*P* > 0.05) concentrations of creatine or its metabolites in the liver, head kidneys, and proximal intestine of HSB. When compared with the 0% glycine group, dietary supplementation with 2% glycine enhanced (*P* < 0.05) concentrations of creatine plus phosphocreatine in the tail kidneys, but dietary supplementation with 1% glycine had no effect. The concentrations of phosphocreatine in the tail kidneys were elevated (*P* < 0.05) by 26% and 44%, respectively, in the 1% and 2% glycine groups, compared with the 0% glycine group. Concentrations of creatinine, guanidinoacetate, and creatine in the tail kidneys did not differ (*P* > 0.05) among the three treatment groups.

Dietary supplementation with 1% and 2% glycine to Phase-II HSB increased (*P* < 0.05) concentrations of creatine, phosphocreatine, and creatine plus phosphocreatine in skeletal muscle in a dose-dependent manner (Table 1). Specifically, when compared with 0% glycine group, 1% and 2% supplemental glycine increased (*P* < 0.05) concentrations of creatine by 14% and 27%, respectively; phosphocreatine by 22% and 39%, respectively; and creatine plus phosphocreatine by 19% and 35%, respectively. In contrast, intramuscular concentrations of creatinine or guanidinoacetate did not differ (*P* > 0.05) among the three groups of fish.

Concentrations of guanidinoacetate in the pancreas of Phase-II HSB were elevated (*P* < 0.05) by 42% in the 2% glycine group, when compared with the 0% glycine group. Concentrations of creatine, phosphocreatine, and creatine plus phosphocreatine in this tissue were increased by supplemental glycine in a dose-dependent manner. Specifically, when compared with the 0% glycine group, dietary supplementation with 1% and 2% glycine increased (*P* < 0.05) concentrations of creatine by 30% and 79%, respectively; phosphocreatine by 67% and 110%, respectively; and creatine plus phosphocreatine by 52% and 97%, respectively. No difference (*P* > 0.05) in pancreatic creatinine concentrations was detected among the three treatment groups.

Dietary supplementation with glycine to Phase-II HSB increased concentrations of guanidinoacetate in the plasma by 15% and 31%, respectively (Table 1). Concentrations of creatine in the plasma did not differ (*P* > 0.05) between the 1% and 2% glycine groups, and values for the glycine-supplemented HSB were 15% higher (*P* < 0.05) than those in the 0% glycine group. No difference (*P* > 0.05) was detected in plasma creatinine concentrations among the three treatment groups. As in Phase-I HSB, phosphocreatine was not detected in the plasma of Phase-II HSB.

#### Effects of dietary glycine supplementation on concentrations of GSH and GSSG in tissues

Data on the concentrations of GSH and GSSG in the tissues of HSB (Phase-II) are summarized in Table 2. When compared with the 0 glycine group, dietary supplementation with 1% and 2% glycine increased (*P* < 0.05) hepatic concentrations of GSH by 11% and 20%, respectively; and GSH plus ½ GSSG by 9% and 17%, respectively. The ratios of GSSG/GSH (mol/mol) in the liver were 10% lower (*P* < 0.05) in the 1% and 2% glycine groups, compared with the 0 glycine group. There were no differences (*P* > 0.05) in hepatic GSSG concentrations among the three treatment groups.

Dietary glycine supplementation influenced GSH concentrations in the head kidneys, tail kidneys, and skeletal muscle of Phase-II HSB in the same pattern (Table 2). No changes (*P* > 0.05) in concentrations of GSH, GSSG, and total glutathione were detected in all these tissues among all treatment groups. Supplemental glycine reduced (*P* < 0.05) the ratios of GSSG/GSH in the head kidneys, tail kidneys, and skeletal muscle by 10%, 11%, and 13%, respectively.

Dietary supplementation with glycine from 0 to 2% increased concentrations of GSH and total glutathione in the proximal intestine of Phase-II HSB in a dose-dependent manner (Table 2). Specifically, compared with the 0% glycine group, dietary supplementation with 1% and 2% glycine increased (*P* < 0.05) concentrations of GSH by 16% and 36%, respectively, and concentrations of total glutathione by 17% and 36%, respectively. Both 1% and 2% glycine supplementations had the same effect in reducing  (*P* < 0.05) the ratio of  intestinal GSSG/GSH by 12%, when compared with the 0% glycine group. There was no difference (*P* > 0.05) in intestinal GSSG concentrations among the three treatment groups. Compared with the 0 glycine group, dietary supplementation with 1% and 2% glycine did not affect (*P* > 0.05) concentrations of GSH, GSSG or total glutathione, or the ratios of GSSG/GSH in the pancreas.

#### Effects of dietary glycine supplementation on the activities of enzymes for creatine synthesis in tissues

Data on the activities of enzymes for creatine synthesis in tissues of Phase-II HSB are summarized in Table 3. Dietary glycine supplementation increased (*P* < 0.05) AGAT activity in the tail kidneys and skeletal muscle in a dose-dependent manner. Specifically, dietary supplementation with 1% and 2% glycine increased (*P* < 0.05) AGAT activities by 24% and 45%, respectively in the tail kidneys, and by 22% and 41%, respectively in the skeletal muscle. AGAT activities in the pancreas were 30% greater (*P* < 0.05) in the 1% and 2% glycine groups, compared with the 0% glycine group. Glycine supplementation did not affect (*P* > 0.05) AGAT activities in the liver, head kidneys, or proximal intestine.

When compared with the 0% glycine group, dietary supplementation with 2% glycine to Phase-II HSB increased (*P* < 0.05) hepatic GAMT activity by 20%, but dietary supplementation with 1% glycine had no effect (*P* > 0.05) (Table 3). Dietary supplementation with 1% and 2% glycine increased (*P* < 0.05) GAMT activity in the skeletal muscle by 18% and 29%, respectively. In contrast, there were no differences (*P* > 0.05) in GAMT activities in the tail kidneys or pancreas among the three treatment groups. Interestingly, GAMT activity was not detected in the head kidneys or proximal intestine in all groups of Phase-II HSB.

#### Effects of dietary glycine supplementation on the activities of GSH-forming enzymes in tissues

Data on the activities of GSH-forming enzymes in the liver and proximal intestine of Phase-II HSB are summarized in Table 4. Dietary supplementation with 1% and 2% glycine increased (*P* < 0.05) hepatic γ-glutamylcysteine synthetase activity in a dose-dependent manner. Specifically, when compared with the 0 glycine group, hepatic γ-glutamylcysteine synthetase activities in the 1% and 2% glycine groups were elevated (*P* < 0.05) by 18% and 37%, respectively. Dietary supplementation with 1% and 2% glycine increased (*P* < 0.05) hepatic GSH synthetase activity to the same extent (by 17%). Compared with the 0% glycine group, dietary supplementation with 2% glycine enhanced (*P* < 0.05) GSH reductase activity in the liver by 14%, but dietary supplementation with 1% glycine had no effect (*P* > 0.05).

In the proximal intestine of Phase-II HSB, activities of GSH reductase and γ-glutamylcysteine synthetase did not differ (*P* > 0.05) between the 1% and 2% glycine groups, but values for the glycine-supplemented HSB were 13% and 17% greater (*P* < 0.05) than those in the 0% glycine group (Table 4). When compared with 0% glycine group, dietary supplementation with 1% and 2% glycine increased (*P* < 0.05) intestinal GSH reductase activities by 17% and 29%, respectively. In the head kidneys, tail kidneys, and skeletal muscle of Phase-II HSB, both 1% and 2% glycine supplementations had the same effect in increasing (*P* < 0.05) the activity of GSH reductase by 38%, 21%, and 45%, respectively (Table 4). Dietary glycine supplementation did not influence (*P* > 0.05) the activities of GSH-synthetic enzymes. In the pancreas, dietary glycine supplementation had no effect (*P* > 0.05) on the activities of these three enzymes for GSH formation.

## Discussion

Creatine and GSH, two metabolites of glycine, are abundant in animal tissues and exert crucial physiological functions by supporting antioxidative capacity and energy metabolism [1]. Because animal growth occurs with the active production of free radicals and ATP consumption, robust antioxidative and energy transformation systems serve as the prerequisite for rapid protein gain and optimal health [25, 26]. Notably, GSH is the most important non-enzymatic antioxidant in animals, whereas creatine helps to store ATP in the form of phosphocreatine for physiological processes such as muscle contractions, protein synthesis, and nutrient transport. Emerging evidence indicates that the endogenous synthesis of glycine may not meet the demand of farmed animals (e.g., HSB and growing pigs) under various nutritional and physiological conditions [13, 14, 27]. Although SBM has a balanced profile for most AAs, this feed ingredient contains much less glycine than fishmeal [19]. Thus, when fishmeal is largely replaced by SBM in aquatic diets, the exogenous supply of glycine will be substantially reduced. The present study evaluated, for the first time to our knowledge, the effects of supplementing glycine to SBM-based diets on the metabolism of GSH and creatine in fish (including HSB) at two different life stages (5–40 g and 110–240 g). Changes in concentrations of creatine and related metabolites, as well as GSH and GSSG, and the activities of creatine- and GSH-forming enzymes in tissues of the HSB are summarized in Supplementary Tables S1, S2, S3, and S4. Future studies are warranted to determine mRNA and protein levels for these enzymes in tissues of glycine-supplemented fish.

### Creatine synthesis by tissues of HSB

The distribution of creatine in tissues of fish appears to be species-specific [7], but major tissues for creatine synthesis in fish remain unknown. Many lower vertebrates including fish, frogs, and birds have both AGAT and GAMT activities in their livers and kidneys, which is in contrast to mammalian and avian species [28, 29]. A zebrafish study demonstrated that AGAT and GAMT were broadly expressed in multiple tissues (central nervous system, liver, and pancreas) in the same pattern as the homologous genes expressed in mammal tissues [30]. A transcriptional study with rainbow trout did not identify a comparable tissue distribution of the expression of genes for creatine synthesis [16]. Instead, the results of this study indicated high abundances of the mRNAs for AGAT, GAMT, and creatine kinase in the skeletal muscle of rainbow trout. The same research group further examined the gene expression of AGAT and GAMT in the liver, kidneys, and skeletal muscle with additional four fish species (maraena whitefish, pikeperch, European perch, and Atlantic herring) and demonstrated that the skeletal muscle possessed the highest abundances of mRNAs for both enzymes among all these tissues. Unfortunately, Borchel et al. [16] did not determine enzymatic activities of AGAT, GAMT or creatine kinase in tissues of the rainbow trout. Such measurements are important because the presence of mRNAs for AGAT and GAMT may not necessarily indicate the presence of their enzymatic activities as shown for pig skeletal muscle [31]. The present study revealed, for the first time, that the liver, tail kidneys, pancreas, and skeletal muscle of HSB had both AGAT and GAMT enzymatic activities (Table 3), with the highest activities of both enzymes in the skeletal muscle. Interestingly, phosphocreatine was not detected in the plasma of both Phase-I and Phase II HSB (Table 1), indicating that the muscle and other tissues of the fish do not release phosphocreatine. These findings suggest that the HSB differs from terrestrial mammals and birds in the major sites of creatine synthesis. In addition, GAMT activity was not detected in the head kidneys (a major immune organ) of HSB, and intestinal GAMT activity was high in 40-g HSB but was undetectable in 240-g HSB (Table 3) due to developmental changes in the tissue-specific expression of enzymes. 

 Like the kidneys and liver of growing pigs [31], increasing the dietary intake of glycine up-regulated the activities of creatine-synthesizing enzymes in the skeletal muscle of HSB (Table 3). In an aqueous environment, fish swim almost constantly, which requires a relatively large amount of energy for muscle movement. The synthesis of creatine from glycine in the skeletal muscle of fish may promote more efficient coupling with local energy metabolism for physiological functions (e.g., constant swimming [32]) when compared with terrestrial mammals and birds, because guanidinoacetate is formed and further converted into creatine in the same muscle fiber. Because activities of creatine-synthesizing enzymes in HSB were upregulated by the dietary intake of glycine, it is crucial that diets must provide sufficient glycine to support creatine synthesis in fish.

### GSH formation in tissues of HSB

Previous research shows that fish species have lower GSH concentrations than mammals and birds, which makes fish more vulnerable to oxidative damage [8]. Unfortunately, fish are constantly exposed to oxidants in the water environment and have a greater challenge by oxidative stress during their lifetime than terrestrial animals. In fish, oxidative stress can be induced by various factors such as heavy metals, poor water quality, temperature changes, predators, and diseases. To maximize the growth potential of fish, many studies have been conducted to improve their weight gains by increasing GSH availability in tissues. For example, supplementing 200 mg/kg of GSH to fishmeal-based diets augmented the growth performance of juvenile Atlantic salmons and upregulated the expression of genes for GSH peroxidase and GSH transferase [33]. Similarly, adding 400 mg/kg of GSH to rapeseed meal-based diets for juvenile grass carp enhanced food intake and weight gain by alleviating liver damage [34]. In addition, supplementing a precursor of GSH (0.2% *N*-acetylcysteine, 0.5% glycine, or both) to plant protein-based diets for Nile tilapia increased growth rate without influencing feed efficiency [35]. The expression of genes for a series of antioxidant-related enzymes (γ-glutamylcysteine synthetase, GSH reductase, glutathione-*S*-transferase, GSH peroxidase, and superoxide dismutase) in diverse tissues was upregulated by these supplements [35]. A more recent study showed that adding 0.5% glycine to plant protein-based diets stimulated growth performance, erythrocyte stability, and mucosal immunity in common carps [36]. Despite a lack of data on GSH concentrations in tissues, these authors proposed that the benefits of dietary glycine supplementation might be mediated by GSH synthesis and antioxidant capacity [36]. However, the above two studies were performed without using the isonitrogenous control in experimental diets.

A novel and important finding from the present study is that dietary glycine supplementation upregulated GSH concentrations as well as the activities of enzymes for GSH formation in the tissues of HSB at two different stages of the life cycle (Table 1 and 2). Consistent with the previous findings, the results of this study revealed that supplementing glycine to SBM-based diets had a positive effect in enhancing the antioxidant capacity of HSB. More specifically, dietary supplementation with glycine helped to maintain a more reducing environment in all examined tissues (the liver, proximal intestine, head kidneys, tail kidneys, and skeletal muscle) except for the pancreas in both Phase-I and Phase-II  HSB. In this regard, adding 1% or 2% glycine to SBM-based had similar effects in reducing GSSG/GSH ratios in tissues. Interestingly, dietary glycine supplementation selectively increased GSH concentrations in the liver and proximal intestine at both life stages (Table 2). Consistently, glycine supplementation upregulated GSH formation  (enzymatic activities of GSH reductase, γ-glutamylcysteine synthetase, and GSH synthetase) in the liver and proximal intestine. These findings indicate that GSH synthesis in these two tissues of fish is very sensitive to regulation by the dietary intake of glycine. Further studies are warranted to determine whether this is also true for glutamate and cysteine (other two precursors of GSH).

### Maximal growth and antioxidative responses of HSB require sufficient creatine and GSH

Requirements of animals for creatine can differ substantially, depending on species, water temperature, stress, and growth rate. Based on the content of water (~ 70%) in the skeletal muscle of HSB, the concentrations of creatine plus phosphocreatine in the skeletal muscle of HSB can be calculated to range from 130 to 165 mmol/L, which are about 3 times those in the skeletal muscle of growing pigs [31]. Additionally, in growing HSB, skeletal muscle (the major reservoir of creatine in the body) accounts for 41% of the total body weight [37]. Thus, the expansion of the muscle for the growth of this fish requires a large amount of creatine. In addition, there is evidence that creatine contributes to osmoregulation in fish for adaptations to changes in the salinities of the ambient water. This ensures the maintenance of whole-body homeostasis that is essential for life in animals including fish. Furthermore, by serving as a component of a major energy buffer system, creatine is crucial for supporting fish-specific burst swimming [32]. Thus, compared with terrestrial farmed animals, creatine may play a more important role in the growth and survival of fish. This view is consistent with results from nutritional studies involving multiple fish species. For example, dietary supplementation with 0.25% creatine improved the growth of spotted seabass fed low-fishmeal diets [38]. The creatine supplementation also promoted energy reserve in the muscle of spotted seabass by increasing ATP concentrations and creatine kinase activity, while inhibiting anaerobic glycolysis by reducing the activities of lactate dehydrogenase and pyruvate kinase [38]. At a salinity of 8 ppt, dietary supplementation with 2% creatine improved the growth performance of channel catfish fry [39]. Interestingly, dietary supplementation with 2% and 4% creatine had no effect on the growth of channel catfish fry at a salinity of 5 ppt but enhanced the weight gain of fish when the salinity of the ambient water increased to 15 ppt [39]. Similar results have been reported for juvenile HSB [40]. In addition, red drum also grew faster when fed diets supplemented with 2% creatine or 1% guanidinoacetate [41], when compared with fish without creatine or guanidinoacetate supplementation. Thus, dietary creatine may interact with salinity to affect the growth of fish. Of interest, an enlarged dorsal muscle area was noted in gilthead seabream fed diets supplemented with 2%, 5%, or 8% creatine [42].

In most nutritional studies with fish, authors did not determine the concentrations of creatine and its metabolites in skeletal muscle and other tissues (e.g., [42–44]). Thus, whether dietary supplementation with creatine increases its availability in tissues of aquatic animals remains unclear. Another novel and salient finding from the current study is that enhancing the dietary intake of glycine increased the enzymatic activities of AGAT and GMAT, as well as the concentrations of both creatine and phosphocreatine in the skeletal muscle of HSB. It is possible that glycine stimulates the expression of genes for AGAT and GMAT in the skeletal muscle of fish, but, to the best of our knowledge, such experimental data are not available for any animal species. Future studies with muscle cell lines isolated from fish are warranted to test this important hypothesis.

An increase in the growth of animals is associated with elevated production of oxidants via tissue-specific metabolism that varies among tissues [45]. Growing-finishing pigs cannot produce sufficient GSH in the liver, kidneys, and small intestine, when fed SBM-based diets [46]. Thus, dietary supplementation with 1% glycine to these animals enhanced this synthetic pathway [15] and whole-body growth [27], while reducing oxidative stress in their tissues [15]. Likewise, dietary supplementation with GSH improves the growth, anti-oxidative capacity, disease resistance and gut morphology in juvenile Atlantic salmon [33], common carp [47], triploid rainbow trout [48], shrimp [49], and crabs [50]. Furthermore, dietary supplementation with 1% or 2% glycine prevented the occurrence of submucosal and lamina propria hemorrhages in the proximal intestine of Phase-I HSB (weighing 5 to 40 g) [13], while improving the intestinal structure of both Phase-I HSB and Phase-II HSB (weighing 110 to 240 g) [14]. Taken together, these findings indicate that the maximal growth, maximal antioxidative responses, and optimal health of fish (including HSB) require sufficient synthesis and availability of GSH. As HSB grow, their requirements for dietary glycine increase possibly due to reduced endogenous synthesis [14]. Besides GSH and creatine syntheses, glycine may up-regulate the expression of genes for anabolic hormones and activate the mechanistic target of rapamycin signaling pathway for stimulating protein synthesis in fish as reported for mammals such as pigs [51, 52]. Based on growth performance and gut health, we recommend that SBM-based diets for Phase-I and Phase-II HSB be supplemented with 1% and 2% glycine, respectively [13, 14]. Because it is not sustainable to feed fish with fish, our findings have important implications for expanding the global production of HSB that are fed low-fishmeal and high-plant ingredient (including SBM) diets.

It is noteworthy that two studies did not identify an effect of dietary glycine supplementation on the growth performance of juvenile rainbow trout [53] or Beluga sturgeon [54]. In the work of Belghit et al. [53], rainbow trout with a mean initial BW of 87 g were restrictively fed (90% of satiety) for 6 weeks a black soldier fly larvae meal (40%)- and corn starch meal (16.48%)-based diet (41% crude protein including 1.73% glycine) supplemented with 0 or 1% glycine. In the investigation of Hoseini et al. [54], Beluga sturgeon were fed for 8 weeks a fishmeal (35%)-, poultry by-product meal (15%)-, wheat meal (23%)-, and soybean meal (25%)-based diet (42% crude protein including 1.8% glycine) supplemented with 0%, 0.25%, 0.5%, or 1% glycine. Neither of the two research groups reported concentrations of GSH or creatine in fish tissues. The discrepancies among the published reports regarding the effects of dietary glycine supplementation on the growth performance of various species of fish may result from differences in a plethora of factors, including AA metabolism (e.g., rates of endogenous synthesis and degradation of glycine), amounts of feed intake per kg BW, dietary nutrient composition, and environmental conditions. For example, restricted feeding [53] and a very low level of dietary cysteine (0.13%) [54] may limit the availability of AAs other than glycine for tissue protein synthesis in rainbow trout and Beluga sturgeon, respectively. For comparison, in our studies, both Phase-I and Phase-II HSB were fed at satiety and the diet contained 0.56% cysteine [13, 14].

## Conclusions

Results of this study indicated that, in contrast to livestock mammals and poultry where creatine synthesis requires the interorgan cooperation of the kidneys and liver but is absent from skeletal muscle, creatine synthesis occurred primarily in skeletal muscles from Phase-I and Phase-II HSB. As in terrestrial mammals and birds, the liver was the most active tissue for GSH synthesis in HSB. In the fish, creatine plus phosphocreatine concentrations in skeletal muscle were 13–15, 13–16, and 112–125 times those in the proximal intestine, kidneys, and liver, respectively, whereas GSH concentrations in the liver were 3.2–3.5, 5.3–6.6, and 9–11 times those in the proximal intestine, kidneys, and skeletal muscle, respectively. Dietary supplementation with glycine improved the antioxidative capacity of fish tissues through increasing the production of both GSH and creatine. Among all the HSB tissues examined, liver and proximal intestine were most sensitive to dietary glycine supplementation with regard to GSH synthesis, as was skeletal muscle regarding creatine synthesis. We conclude that SBM-based diets did not provide adequate glycine for the syntheses of creatine and GSH in growing Phase-I or Phase-II HSB and that dietary supplementation with 1% or 2% glycine is necessary for the maximal growth and feed efficiency, as well as the optimal health (including intestinal health) of these aquatic animals. Based on the metabolic and growth data, glycine is a conditionally essential amino acid for HSB. Future studies are warranted to determine effects of dietary glycine supplementation on muscle protein turnover and its associated regulatory pathways, as well as the molecular regulation of the expression of genes for GSH and creatine syntheses at transcriptional and translational levels.

### Supplementary Information

**Additional file 1: Table S1. **Changes in concentrations of creatine and related metabolites in tissues of hybrid striped bass fed a soybean meal (58%)-based diet supplemented with 0, 1%, or 2% glycine. **Table S2. **Changes in concentrations of glutathione (GSH) and glutathione disulfide (GSSG) in tissues of hybrid striped bass fed a soybean meal (58%)-based diet supplemented with 0, 1%, or 2% glycine.** Table S3. **Changes in activities of creatine-synthetic enzymes in tissues of hybrid striped bass fed a soybean meal (58%)-based diet supplemented with 0, 1%, or 2% glycine.** Table S4. **Changes in activities of glutathione-forming enzymes in tissues of hybrid striped bass fed a soybean meal (58%)-based diet supplemented with 0, 1%, or 2% glycine.

## Data Availability

All data generated or analyzed during this study are available from the corresponding author upon reasonable request.

## Abbreviations

AA: Amino acid
AGAT: L-arginine:glycine amidinotransferase
GAMT: Guanidinoacetate *N*-methyltransferase
GSH: Glutathione (reduced form)
GSSG: Glutathione disulfide (oxidized glutathione)
HPLC: High-performance liquid chromatography
HSB: Hybrid striped bass
SBM: Soybean meal

## References

[1] Wu G (2022). Amino acids: biochemistry and nutrition.

[2] Wu G, Li P (2022). The, “ideal protein” concept is not ideal in animal nutrition. Exp Biol Med.

[3] Li XY, Zheng SX, Wu G (2021). Nutrition and functions of amino acids in fish. Adv Exp Med Biol..

[4] Sukhovskaya IV, Borvinskaya EV, Smirnov LP (2017). Role of glutathione in functioning of the system of antioxidant protection in fish (review). Inland Water Biol.

[5] Wu G, Fang YZ, Yang S, Lupton JR, Turner ND (2004). Glutathione metabolism and its implications for health. J Nutr.

[6] Brosnan JT, da Silva RP, Brosnan ME (2011). The metabolic burden of creatine synthesis. Amino Acids.

[7] Hunter A (1929). The creatine content of the muscles and some other tissues in fishes. J Biol Chem.

[8] Leggatt RA. Glutathione in fish: transport, influence of temperature and growth rate, and interaction with the stress response. Thesis. University of British Columbia, Vancouver, Canada. 2006.

[9] Hissen KL, He WL, Wu G, Criscitiello MF (2023). Immunonutrition: facilitating mucosal immune response in teleost intestine with amino acids through oxidant-antioxidant balance. Front Immunol.

[10] Harrell R. Cultured aquatic species information programme - Morone hybrid (genus *Morone*, hybrids). In: Fisheries and Aquaculture. Rome: Food and Agriculture Organization of the United Nations. 2016; pp. 1–19.

[11] USDA. USDA/ARS National Program 106 Aquaculture Action Plan 2020–2024. Agricultural Research Service, the United States Department of Agriculture, Washington, DC. 2019. https://www.ars.usda.gov/ARSUserFiles/np106/NP106%20Aquaculture%20Action%20Plan%202020-2024%20Amended%207-20-2021_final2.pdf. Accessed 5 Mar 2024.

[12] Webster CD, Muzinic LA, Thompson KR. Hybrid striped bass culture a U.S. success story. Global Aquaculture Advocate. 2020. www.globalseafood.org. Accessed 5 Mar 2024.

[13] Li XY, He WL, Wu G (2023). Dietary glycine supplementation enhances the growth performance of hybrid striped bass (*Morone saxatilis ♀× Morone chrysops ♂*) fed soybean meal-based diets. J Anim Sci..

[14] He WL, Li XY, Wu G (2023). Dietary glycine supplementation improves the growth performance of 110- to 240-g (Phase-II) hybrid striped bass (*Morone saxatilis* ♀× *Morone chrysops* ♂) fed soybean meal-based diets. J Anim Sci..

[15] He WL. Role of dietary glycine in the growth of pigs (*Sus scrofa*) and hybrid striped bass (*Morone saxatilis ****♀***** ×** *Morone chrysops* **♂**). PhD Dissertation, Texas A&M University, College Station, Texas, USA. 2023.

[16] Borchel A, Verleih M, Rebl A, Kühn C, Goldammer T (2014). Creatine metabolism differs between mammals and rainbow trout (*Oncorhynchus mykiss*). Springerplus.

[17] National Research Council (NRC) (2011). Nutrient Requirements of Fish and Shrimp.

[18] Hou YQ, Li XL, Dai ZL, Wu ZL, Bazer FW, Wu G (2018). Analysis of glutathione in biological samples by HPLC involving pre-column derivatization with *o*-phthalaldehyde. Methods Mol Biol.

[19] Li P, Wu G (2020). Composition of amino acids and related nitrogenous nutrients in feedstuffs for animal diets. Amino Acids.

[20] Verhoeven NM, Roos B, Struys EA, Salomons GS, van der Knaap MS, Cornelis JC (2004). Enzyme assay for diagnosis of guanidinoacetate methyltransferase deficiency. Clin Chem.

[21] Carducci C, Birarelli M, Santagata P, Leuzzi V, Carducci C, Antonozzi I (2001). Automated high-performance liquid chromatographic method for the determination of guanidinoacetic acid in dried blood spots: a tool for early diagnosis of guanidinoacetate methyltransferase deficiency. J Chromatogr B.

[22] Nardi G, Cipollaro M (1990). Assay of y-glutamylcysteine synthetase and glutathione synthetase in erythrocytes by high-performance liquid chromatography with fluorimetric detection. J Chromatogr.

[23] Goldberg DM, Spooner RJ, Bergmeyer HU (1983). Glutathione reductase. Methods of Enzymatic Analysis.

[24] Gerald KB (1990). Common multiple comparison procedures. Nurse Anesth.

[25] Wu G (2009). Amino acids: metabolism, functions, and nutrition. Amino Acids.

[26] Wang W, Wu Z, Lin G, Hu S, Wang B, Dai Z (2014). Glycine stimulates protein synthesis and inhibits oxidative stress in pig small intestinal epithelial cells. J Nutr.

[27] He WL, Posey EA, Steele CC, Savell JW, Bazer FW, Wu G (2023). Dietary glycine supplementation enhances postweaning growth and meat quality of pigs with intrauterine growth restriction. J Anim Sci..

[28] Van Pilsum JF, Stephens GC, Andtaylor D (1972). Distribution of creatine, guanidinoacetate and the enzymes for their biosynthesis in the animal kingdom. Biochem J.

[29] Walker JB (1979). Creatine: biosynthesis, regulation, and function. Adv Enzymol.

[30] Wang L, Chen D, Yang L, Huang S, Zhang Y, Zhang H (2010). Expression patterns of the creatine metabolism-related molecules AGAT, GAMT and CT1 in adult zebrafish *Danio rerio*. J Fish Biol.

[31] Posey EA, He WL, Wu G (2021). Dietary glycine supplementation during growing and finishing phases increases tissue concentrations of total creatine and gene expression of creatine-synthetic enzymes in low-birthweight pigs. J Anim Sci.

[32] Wuertzm S, Reiser S (2023). Creatine: a valuable supplement in aquafeeds?. Rev Aquac.

[33] Ma J, Zhang J, Sun GX, Lou YN, Li Y (2019). Effects of dietary reduced glutathione on the growth and antioxidant capacity of juvenile Atlantic salmon (*Salmo salar*). Aquac Nutr.

[34] Yuan XC, Zhou Y, Liang XF, Guo X, Fang L, Li J (2014). Effect of dietary glutathione supplementation on the biological value of rapeseed meal to juvenile grass carp, *Ctenopharyngodon*
*idellus*. Aquac Nutr.

[35] Xie S, Zhou W, Tian L, Niu J, Liu Y (2016). Effect of N-acetyl cysteine and glycine supplementation on growth performance, glutathione synthesis, anti-oxidative and immune ability of Nile tilapia, Oreochromis niloticus. Fish Shellfish Immunol.

[36] Abbasi M, Taheri Mirghaed A, Hoseini SM, Rajabiesterabadi H, Hoseinifar SH, Van Doan H (2023). Effects of dietary glycine supplementation on growth performance, immunological, and erythrocyte antioxidant parameters in common carp, Cyprinus carpio. Animals (Basel).

[37] Li XY, Zheng SX, Wu G (2021). Nutrition and functions of amino acids in fish. Adv Exp Med Biol.

[38] Lin JB, Liao YQ, Li XS, Lu KL, Song K, Wang L (2023). Effects of dietary creatine levels on the growth, muscle energy metabolism and meat quality of spotted seabass (*Lateolabrax maculatus*) fed low-fishmeal diets. Aquaculture.

[39] Burns AF. Effects of swimming and creatine supplementation on cultured fishes. PhD Dissertation. Texas A&M University, College Station. 2019.

[40] Burns AF, Gatlin DM (2022). Effects of dietary creatine on juvenile hybrid striped bass in low-salinity and brackish waters. J World Aquac Soc.

[41] Stites W, Wang L, Gatlin DM (2020). Evaluation of dietary creatine and guanidinoacetic acid supplementation in juvenile red drum *Sciaenops ocellatus*. Aquac Nutr.

[42] Ramos-Pinto L, Lopes G, Sousa V, Castro LFC, Schrama D, Rodrigues P (2019). Dietary creatine supplementation in gilthead seabream (*Sparus aurata*) increases dorsal muscle area and the expression of myod1 and capn1 genes. Front Endocrinol (Lausanne).

[43] Tian JJ, Li YP, Xia Y, Zhang K, Li ZF, Gong WB (2022). Dietary creatine reduces lipid accumulation by improving lipid catabolism in the herbivorous grass carp, Ctenopharyngodon Idella Aquac. Nutr.

[44] Janes D, Suehs B, Gatlin DM (2023). Dietary creatine and guanidinoacetic acid supplementation have limited effects on hybrid striped bass. Fish Physiol Biochem.

[45] Wu G (2018). Principles of Animal Nutrition.

[46] Fan X, Li S, Wu Z, Dai Z, Li J, Wang X (2019). Glycine supplementation to breast-fed piglets attenuates post-weaning jejunal epithelial apoptosis: a functional role of CHOP signaling. Amino Acids.

[47] Xue S, Chen S, Ge Y, Guan T, Han Y (2022). Regulation of glutathione on growth performance, biochemical parameters, non-specific immunity, and related genes of common carp (Cyprinus carpio) exposed to ammonia. Aquaculture.

[48] Wang C, Su B, Lu S, Han S, Jiang H, Li Z (2021). Effects of glutathione on growth, intestinal antioxidant capacity, histology, gene expression, and microbiota of juvenile triploid *Oncorhynchus mykiss*. Front Physiol.

[49] Wang X, Xu W, Zhou H, Zhang Y, Gao W, Zhang W (2018). Reduced glutathione supplementation in practical diet improves the growth, anti-oxidative capacity, disease resistance and gut morphology of shrimp *Litopenaeus vannamei*. Fish Shellfish Immunol.

[50] Liu JD, Liu WB, Zhang DD, Xu CY, Zhang CY, Zheng XC (2020). Dietary reduced glutathione supplementation can improve growth, antioxidant capacity, and immunity on Chinese mitten crab, Eriocheir sinensis. Fish Shellfish Immunol.

[51] Sun K, Wu Z, Ji Y, Wu G (2016). Glycine regulates protein turnover by activating protein kinase B/mammalian target of rapamycin and by inhibiting MuRF1 and atrogin-1 gene expression in C2C12 myoblasts. J Nutr.

[52] Liu Y, Wang X, Wu H, Chen S, Zhu H, Zhang J (2016). Glycine enhances muscle protein mass associated with maintaining Akt-mTOR-FOXO1 signaling and suppressing TLR4 and NOD2 signaling in piglets challenged with LPS. Am J Physiol..

[53] Belghit I, Philip AJP, Maas RM, Lock E-J, Eding EH, Espe M, Schrama JW (2023). Impact of dietary glutamate and glycine on growth and nutrient utilization in rainbow trout (*Oncorhynchus mykiss*). Aquaculture.

[54] Hoseini SM, Moghaddam AA, Ghelichpour M, Pagheh E, Haghpanah A, Gharavi B (2022). Dietary glycine supplementation modulates antioxidant and immune responses of beluga, *Huso huso, juveniles*. Aquac Rep.

