# Supplementary material for: Dietary glycine supplementation enhances syntheses of creatine and glutathione by tissues of hybrid striped bass (Morone saxatilis ♀ × Morone chrysops ♂) fed soybean meal-based diets
Source: J Anim Sci Biotechnol. 2024 May 9;15:67. doi: 10.1186/s40104-024-01024-5 (PMC11080189; doi:10.1186/s40104-024-01024-5)
Supplement: Supplementary file 1 — Additional file 1: Table S1. Changes in concentrations of creatine and related metabolites in tissues of hybrid striped bass fed a soybean meal (58%)-based diet supplemented with 0, 1%, or 2% glycine. Table S2. Changes in concentrations of glutathione (GSH) and glutathione disulfide (GSSG) in tissues of hybrid striped bass fed a soybean meal (58%)-based diet supplemented with 0, 1%, or 2% glycine. Table S3. Changes in activities of creatine-synthetic enzymes in tissues of hybrid striped bass fed a soybean meal (58%)-based diet supplemented with 0, 1%, or 2% glycine. Table S4. Changes in activities of glutathione-forming enzymes in tissues of hybrid striped bass fed a soybean meal (58%)-based diet supplemented with 0, 1%, or 2% glycine. [file 40104_2024_1024_MOESM1_ESM.pdf]

Supplementary Table S1 Changes in concentrations of creatine and related metabolites in tissues of hybrid striped bass fed a soybean meal (58%)-based diet supplemented with 0, 1, or 2% glycine

Variable	Tissue or plasma	Response to dietary supplementation with 0%, 1%, and 2% glycine
		**Phase-I (5 to 40 g) hybrid striped bass**
Creatine	Skeletal muscle	↑ (1% or 2% glycine vs 0% glycine); "─" between 1% and 2% glycine groups
	Liver	↑ (1% or 2% glycine vs 0% glycine); "─" between 1% and 2% glycine groups
	Kidney	↑ (in a dose-dependent manner)
	Proximal intestine	"─" among the three groups of fish
	Plasma	↑ (1% or 2% glycine vs 0% glycine); "─" between 1% and 2% glycine groups
Phosphocreatine	Skeletal muscle	↑ (1% or 2% glycine vs 0% glycine); "─" between 1% and 2% glycine groups
	Liver	"─" among the three groups of fish
	Kidney	"─" among the three groups of fish
	Proximal intestine	"─" among the three groups of fish
	Plasma	Phosphocreatine was not detected.
Guanidinoacetate	Skeletal muscle	↑ (in a dose-dependent manner)
	Liver	↑ (2% glycine vs 0% glycine); "─" between 1% and 0 or 2% glycine groups
	Kidney	↑ (in a dose-dependent manner)
	Proximal intestine	↑ (2% glycine vs 0% glycine); "─" between 1% and 0 or 2% glycine groups
	Plasma	↑ (in a dose-dependent manner)
Creatinine	All tissues studied	"─" among the three groups of fish
		**Phase-II (110 to 240 g) hybrid striped bass**
Creatine	Skeletal muscle	↑ (in a dose-dependent manner)
	Liver	"─" among the three groups of fish
	Head kidney	"─" among the three groups of fish
	Tail kidney	"─" among the three groups of fish
	Pancreas	↑ (in a dose-dependent manner)
	Proximal intestine	"─" among the three groups of fish
	Plasma	↑ (1% or 2% glycine vs 0% glycine); "─" between 1% and 2% glycine groups
Phosphocreatine	Skeletal muscle	↑ (in a dose-dependent manner)
	Liver	"─" among the three groups of fish
	Head kidney	"─" among the three groups of fish
	Tail kidney	↑ (1% or 2% glycine vs 0% glycine); "─" between 1% and 2% glycine groups
	Pancreas	↑ (in a dose-dependent manner)
	Proximal intestine	"─" among the three groups of fish
	Plasma	Phosphocreatine was not detected.
Guanidinoacetate	Skeletal muscle	"─" among the three groups of fish
	Liver	Guanidinoacetate was not detected.
	Head kidney	Guanidinoacetate was not detected.
	Tail kidney	"─" among the three groups of fish
	Pancreas	↑ (2% glycine vs 0% glycine); "─" between 1% and 0 or 2% glycine groups
	Proximal intestine	"─" among the three groups of fish
	Plasma	↑ (in a dose-dependent manner)
Creatinine	All tissues studied	"─" among the three groups of fish

↑, Increase; "─", no change.

Supplementary Table S2 Changes in concentrations of glutathione (GSH) and glutathione disulfide (GSSG) in tissues of hybrid striped bass fed a soybean meal (58%)-based diet supplemented with 0, 1, or 2% glycine

Variable	Tissue or plasma	Response to dietary supplementation with 0%, 1%, and 2% glycine
		**Phase-I (5 to 40 g) hybrid striped bass**
GSH	Skeletal muscle	"─" among the three groups of fish
	Liver	↑ (1% or 2% glycine vs 0% glycine); "─" between 1% and 2% glycine groups
	Kidney	"─" among the three groups of fish
	Proximal intestine	↑ (in a dose-dependent manner)
GSSG	Skeletal muscle	↓ (1% or 2% glycine vs 0% glycine); "─" between 1% and 2% glycine groups
	Liver	"─" among the three groups of fish
	Kidney	↓ (1% or 2% glycine vs 0% glycine); "─" between 1% and 2% glycine groups
	Proximal intestine	↑ (in a dose-dependent manner)
GSSG/GSH (mol/mol)	Skeletal muscle	↓ (1% or 2% glycine vs 0% glycine); "─" between 1% and 2% glycine groups
	Liver	↓ (1% or 2% glycine vs 0% glycine); "─" between 1% and 2% glycine groups
	Kidney	↓ (1% or 2% glycine vs 0% glycine); "─" between 1% and 2% glycine groups
	Proximal intestine	↓ (1% or 2% glycine vs 0% glycine); "─" between 1% and 2% glycine groups
Total glutathione[1]	Skeletal muscle	"─" among the three groups of fish
	Liver	↑ (1% or 2% glycine vs 0% glycine); "─" between 1% and 2% glycine groups
	Kidney	"─" among the three groups of fish
	Proximal intestine	↑ (in a dose-dependent manner)
		**Phase-II (110 to 240 g) hybrid striped bass**
GSH	Skeletal muscle	"─" among the three groups of fish
	Liver	↑ (in a dose-dependent manner)
	Head kidney	"─" among the three groups of fish
	Tail kidney	"─" among the three groups of fish
	Pancreas	"─" among the three groups of fish
	Proximal intestine	↑ (in a dose-dependent manner)
GSSG	Skeletal muscle	"─" among the three groups of fish
	Liver	"─" among the three groups of fish
	Head kidney	"─" among the three groups of fish
	Tail kidney	"─" among the three groups of fish
	Pancreas	"─" among the three groups of fish
	Proximal intestine	"─" among the three groups of fish
GSSG/GSH (mol/mol)	Skeletal muscle	↓ (1% or 2% glycine vs 0% glycine); "─" between 1% and 2% glycine groups
	Liver	↓ (1% or 2% glycine vs 0% glycine); "─" between 1% and 2% glycine groups
	Head kidney	↓ (1% or 2% glycine vs 0% glycine); "─" between 1% and 2% glycine groups
	Tail kidney	↓ (1% or 2% glycine vs 0% glycine); "─" between 1% and 2% glycine groups
	Pancreas	"─" among the three groups of fish
	Proximal intestine	↓ (1% or 2% glycine vs 0% glycine); "─" between 1% and 2% glycine groups
Total glutathione[1]	Skeletal muscle	"─" among the three groups of fish
	Liver	↑ (in a dose-dependent manner)
	Head kidney	"─" among the three groups of fish
	Tail kidney	"─" among the three groups of fish
	Pancreas	"─" among the three groups of fish
	Proximal intestine	↑ (in a dose-dependent manner)

↑, Increase; ↓, Decrease; "─", no change.
1 Glutathione + ½ glutathione disulfide.

Supplementary Table S3 Changes in activities of creatine-synthetic enzymes in tissues of hybrid striped bass fed a soybean meal (58%)-based diet supplemented with 0, 1, or 2% glycine

Variable	Tissue or plasma	Response to dietary supplementation with 0%, 1%, and 2% glycine
		**Phase-I (5 to 40 g) hybrid striped bass**
AGAT	Skeletal muscle	↑ (in a dose-dependent manner)
	Liver	"─" among the three groups of fish
	Kidney	↑ (in a dose-dependent manner)
	Proximal intestine	"─" among the three groups of fish
GAMT	Skeletal muscle	↑ (1% or 2% glycine vs 0% glycine); "─" between 1% and 2% glycine groups
	Liver	↑ (2% glycine vs 0% glycine); "─" between 1% and 0 or 2% glycine groups
	Kidney	"─" among the three groups of fish
	Proximal intestine	"─" among the three groups of fish
		**Phase-II (110 to 240 g) hybrid striped bass**
AGAT	Skeletal muscle	↑ (in a dose-dependent manner)
	Liver	"─" among the three groups of fish
	Head kidney	"─" among the three groups of fish
	Tail kidney	↑ (in a dose-dependent manner)
	Pancreas	↑ (1% or 2% glycine vs 0% glycine); "─" between 1% and 2% glycine groups
	Proximal intestine	"─" among the three groups of fish
GAMT	Skeletal muscle	↑ (1% or 2% glycine vs 0% glycine); "─" between 1% and 2% glycine groups
	Liver	↑ (2% glycine vs 0% glycine); "─" between 1% and 0 or 2% glycine groups
	Head kidney	Enzymatic activity was not detected.
	Tail kidney	"─" among the three groups of fish
	Pancreas	"─" among the three groups of fish
	Proximal intestine	Enzymatic activity was not detected.

↑, Increase; "─", no change.
AGAT, arginine:glycine amidinotransferase; GAMT, guanidinoacetate methyltransferase.

Supplementary Table S4 Changes in activities of glutathione-forming enzymes in tissues of hybrid striped bass fed a soybean meal (58%)-based diet supplemented with 0, 1, or 2% glycine

Variable	Tissue or plasma	Response to dietary supplementation with 0%, 1%, and 2% glycine
		**Phase-I (5 to 40 g) hybrid striped bass**
GCS	Skeletal muscle	"–" among the three groups of fish
	Liver	↑ (in a dose-dependent manner)
	Kidney	"–" among the three groups of fish
	Proximal intestine	↑ (1% or 2% glycine vs 0% glycine); "–" between 1% and 2% glycine groups
Glutathione synthetase	Skeletal muscle	"–" among the three groups of fish
	Liver	↑ (in a dose-dependent manner)
	Kidney	"–" among the three groups of fish
	Proximal intestine	↑ (1% or 2% glycine vs 0% glycine); "–" between 1% and 2% glycine groups
Glutathione reductase	Skeletal muscle	↑ (1% or 2% glycine vs 0% glycine); "–" between 1% and 2% glycine groups
	Liver	↑ (2% glycine vs 0% or 1% glycine); "–" between 1% and 0% glycine groups
	Kidney	↑ (1% or 2% glycine vs 0% glycine); "–" between 1% and 2% glycine groups
	Proximal intestine	↑ (1% or 2% glycine vs 0% glycine); "–" between 1% and 2% glycine groups
		**Phase-II (110 to 240 g) hybrid striped bass**
GCS	Skeletal muscle	"–" among the three groups of fish
	Liver	↑ (in a dose-dependent manner)
	Head kidney	"–" among the three groups of fish
	Tail kidney	"–" among the three groups of fish
	Pancreas	"–" among the three groups of fish
	Proximal intestine	↑ (1% or 2% glycine vs 0% glycine); "–" between 1% and 2% glycine groups
Glutathione synthetase	Skeletal muscle	"–" among the three groups of fish
	Liver	↑ (1% or 2% glycine vs 0% glycine); "–" between 1% and 2% glycine groups
	Head kidney	"–" among the three groups of fish
	Tail kidney	"–" among the three groups of fish
	Pancreas	"–" among the three groups of fish
	Proximal intestine	↑ (in a dose-dependent manner)
Glutathione reductase	Skeletal muscle	↑ (1% or 2% glycine vs 0% glycine); "–" between 1% and 2% glycine groups
	Liver	↑ (2% glycine vs 0% or 1% glycine); "–" between 1% and 0% glycine groups
	Head kidney	↑ (1% or 2% glycine vs 0% glycine); "–" between 1% and 2% glycine groups
	Tail kidney	↑ (1% or 2% glycine vs 0% glycine); "–" between 1% and 2% glycine groups
	Pancreas	"–" among the three groups of fish
	Proximal intestine	↑ (1% or 2% glycine vs 0% glycine); "–" between 1% and 2% glycine groups

↑, Increase; "–", no change.
GCS, γ-glutamylcysteine synthetase.
